# Influence of Molybdenum on the Microstructure, Mechanical Properties and Corrosion Resistance of Ti_20_Ta_20_Nb_20_(ZrHf)_20−x_Mo_x_ (Where: x = 0, 5, 10, 15, 20) High Entropy Alloys

**DOI:** 10.3390/ma15010393

**Published:** 2022-01-05

**Authors:** Karsten Glowka, Maciej Zubko, Paweł Świec, Krystian Prusik, Magdalena Szklarska, Dariusz Chrobak, János L. Lábár, Danuta Stróż

**Affiliations:** 1 Institute of Materials Engineering, University of Silesia in Katowice, 75 Pułku Piechoty 1A St., 41-500 Chorzow, Poland; karsten.glowka@us.edu.pl (K.G.); pawel.swiec@us.edu.pl (P.Ś.); krystian.prusik@us.edu.pl (K.P.); magdalena.szklarska@us.edu.pl (M.S.); dariusz.chrobak@us.edu.pl (D.C.); danuta.stroz@us.edu.pl (D.S.); 2 Department of Physics, Faculty of Science, University of Hradec Králové, Rokitanského 62, 50003 Hradec Kralove, Czech Republic; 3 Centre for Energy Research, Institute for Technical Physics and Materials Science, Konkoly Thege Miklós út 29-33, H-1121 Budapest, Hungary; labar.janos@ek-cer.hu

**Keywords:** multi-component alloys, high entropy alloys, microstructure analysis, mechanical properties, corrosion resistance

## Abstract

The presented work was focused on investigating the influence of the (hafnium and zirconium)/molybdenum ratio on the microstructure and properties of Ti_20_Ta_20_Nb_20_(ZrHf)_20__−x_Mo_x_ (where: x = 0, 5, 10, 15, 20 at.%) high entropy alloys in an as-cast state. The designed chemical composition was chosen due to possible future biomedical applications. Materials were obtained from elemental powders by vacuum arc melting technique. Phase analysis revealed the presence of dual body-centered cubic phases. X-ray diffraction showed the decrease of lattice parameters of both phases with increasing molybdenum concentration up to 10% of molybdenum and further increase of lattice parameters. The presence of two-phase matrix microstructure and hafnium and zirconium precipitates was proved by scanning and transmission electron microscopy observation. Mechanical property measurements revealed decreased micro- and nanohardness and reduced Young’s modulus up to 10% of Mo content, and further increased up to 20% of molybdenum addition. Additionally, corrosion resistance measurements in Ringers’ solution confirmed the high biomedical ability of studied alloys due to the presence of stable oxide layers.

## 1. Introduction

Since the dawn of time, people have modified metallic materials by adding new alloying elements and have developed new methods for producing and processing materials. There is a strong correlation between the physical and chemical properties of alloying elements on the functional properties of the solidified metals. The first produced binary alloys were mainly composed of dominant metallic elements and one alloying element [1]. However, more advanced engineering materials containing more alloying elements in their microstructures were also produced [2]. Novel materials produced by innovative methods consisting of more than two alloying elements belonged to the group of multicomponent alloys [3].

Further increase in the number of alloying elements led to the discovery of various alloys with superior properties, such as Ni-based superalloys containing up to 10 precisely chosen chemical elements with various concentrations [4]. The idea of multi-component alloys led to the new concept of materials containing many chemical elements with equiatomic ratios, such as high entropy alloys (HEAs) representing multi-principal-element materials. High entropy alloys are defined as alloys that are composed of at least five chemical elements in equiatomic or near-equiatomic ratios. While the studies on HEAs started in the late 1970s, the first high entropy alloy was reported by Cantor et al. in 2004 [5,6]. Five-elemental, equiatomic Co_20_Cr_20_Fe_20_Mn_20_Ni_20_ Cantor’s alloy exhibited single-phase, faced-centered cubic (FCC) structure and dendritic microstructure [5].

Moreover, in the same year, an independent study of nanostructure high entropy alloy performed by Yeh et al. was also reported [7]. Two definitions of HEAs based on chemical composition and configurational entropy (ΔS_conf_) have been proposed [7]. Up to now, the number of reported high entropy alloy compositions is still increasing. Since 2019, 918 publications have been reported; however, only 146 designed HEA compositions exhibited single-phase structures [8]. Based on the presented results, it can be denoted that multi-phase structures mainly form for a large group of high entropy alloys [8]. Moreover, HEAs exhibit improved properties in comparison to conventional materials [9]. Due to the excellent properties, high entropy alloys can find industrial application, e.g., as 3D printing (AlCoCrFeNi, AlCoFeNiTiVZr) [10,11,12], functional (Co_25_Mo_45_Fe_10_Ni_10_Cu_10_) or catalytic (PtPdRhRuCe) usages [13]. High entropy composition FeCoNiAl_0.2_Si_0.2_ designed and studied by Zuo et al. was proposed as a good candidate for a soft magnetic material [13]. On the other hand, multi-component (AgCu)(InGa)Te_2_ and Cu_8_Ge(SeTe)_6_ HEAs also exhibit good thermoelectrical properties [14]. It is also worth emphasizing that high entropy films can also exhibit good solar absorption properties, which can be applied in the solar cell manufacturing process, such as NbTiAlSiWN described by Sheng et al. [15]. CrMnFeCoNi HEAs also revealed suitability in cryogenic applications at liquid nitrogen temperatures [16]. High entropy materials, e.g., TiZrHfCoNiCu and CoNiCuAlGaIn, can also exhibit shape memory effect (SME), whereas for other NiTi-based HEAs (Ni_20_Ti_20_Ta_20_Co_20_Cu_20_ or Ni_35_Ti_35_Ta_10_Co_10_Cu_10_), such properties are expected [17,18,19]. Refractory properties at elevated and high temperatures of high entropy materials should also be taken into account. Refractory high entropy alloys (RHEAs) mainly studied by Senkov et al. are composed of high melting point elements such as Ta, W, Mo, etc., for example, MoNbTaVW [20]. Another example of RHEAs is novel, low-density TiAlVNbMo or precipitate hardened NiCoFeCrAlTi alloys, respectively [21,22]. It should be underlined that HEAs also exhibit high potential ability for biomedical applications [23]. As is well known, materials proposed for biomedical applications belong to a particular group of engineering materials and should consist only of biocompatible elements. Additionally, these materials have to achieve other strict requirements such as non-allergic character, and a high level of oxidation resistance in the human body fluid environment. Furthermore, biomedical materials designed for a specific application, such as bone implants, should also exhibit Young’s modulus close to human bones [24]. Up to now, metallic engineering materials have been the most widely used for biomedical applications. However, ceramic materials and polymers are also widely applied in medicine [25]. The newest data revealed that some conducting polymers could also be applied in drug delivery systems in the neurodegenerative disease treatment process, which was reported in the literature by Krawczyk et al. [26].

As was mentioned earlier, biomedical alloys may contain only biocompatible elements including Ti, Nb, Zr, Ta. However, the biocompatibility of chemical elements such as Mo and Hf was denoted as discussed and unknown [24]. Moreover, all the above-mentioned biocompatible elements can be classified as β-phase stabilizing elements in Ti-based alloys [27]. Molybdenum could be used in Ti-based alloys designed for biomedical applications, such as TiNbMoSn and TiTaMo obtained by powder metallurgy technique [28,29,30]. Additionally, biocompatible properties of pure Hf have also been investigated. In vivo studies of pure hafnium described by Matsuno et al. revealed good biocompatibility and osteogenesis [31]. Additionally, rat and rabbit tissue response to hafnium was carried out by Mohammadi et al. [32]. The studies revealed a similar reaction in soft and hard tissues, which suggests that pure hafnium could be used as a potential element for biomedical alloys [32]. In 2014 Sin et al. performed corrosion and tribocorrosion measurements of pure Hf in simulated body fluid mixtures. The results confirmed good corrosion resistance and quick repassivation of the oxide layer after damage [33].

Up to now, Ti-based alloys are mainly used as biomaterials due to the excellent biocompatibility properties of pure Ti such as non-carcinogenicity, non-genotoxicity, non-mutagenicity, non-allergic and also revealing a high level of corrosion resistance [24,34,35].

High entropy alloys also exhibit high biomedical application ability. However, the newest literature data confirmed that only a small group of HEAs were described and proposed as potential biomaterials. Li et al. proposed TiNbZrTaSi composition obtained by spark plasma sintering process. Due to good biocompatibility, low cost and low density silicon addition was chosen [36]. Popescu et al. obtained by mechanical alloying (MA) a promising multi-phase structure TiZrNbTaFe biomaterial alloy [37]. Five-elemental, equiatomic, Mo-containing, high entropy alloys TiZrNbTaMo were designed for orthopedic implant application. Dual body-centered cubic (BCC) solid solutions with slight differences in lattice parameters have been observed. Moreover, the presence of dendritic and interdendritic regions was also observed. Elemental segregation was closely correlated with differences in melting points of all alloying elements. Additionally, high entropy TiZrNbTaMo alloy shows excellent corrosion resistance measured in simulated physiological environment medium [38]. On the other hand, studies for similar chemical composition (TiZrNbTaMo) were performed by Todai et al. [39]. HEAs obtained by vacuum arc melting followed by annealing also revealed dual-BCC solid solutions with slight differences in lattice parameters [38]. For similar high entropy composition, microstructure analysis also revealed dendritic and interdendritic regions [38,39]. Equiatomic and non-equiatomic TiNbTaZrMo high-entropy alloy compositions for biomaterials were also studied and reported by Hori et al. [40]. Samples were obtained by vacuum arc melting. XRD phase analysis revealed dual-BCC structures. Microstructure analysis also revealed the presence of dendritic and interdendritic regions. Mechanical properties confirmed higher proof stress (σ_0.2_) for studied equiatomic HEA compositions. Biocompatibility measurements confirmed better osteoblast adhesion for high entropy compositions (TiNbTaZrMo) in comparison to SUS316 L and cp-Ti materials [40]. Due to the improvement of mechanical properties, the TiNbTaZrMo bio-high entropy alloys (bio-HEAs) were also produced by the selective laser melting (SLM) technique [41]. The presence of BCC solid solution for atomized powder and SLM product has been confirmed. Additionally, a printed product’s microstructure analysis revealed coarse and fine grain structures. Mechanical properties have been determined compared to Ti_1.4_Nb_0.6_Ta_0.6_Zr_1.4_Mo_0.6_ bio-HEA obtained via vacuum arc melting technique and are reported in the literature by Hori et al. [40]. For SLM product proof stress was much higher than non-equiatomic, as-cast bio-HEA. Additionally, the measured Youngs modulus for the SLM product was similar to metallic biomaterials such as cp-Ti, Ti6Al4V and 316 L medical stainless steel. Moreover, biocompatibility properties of the SLM product confirmed the highest cell density compared to cp-Ti, 316 L SS and near-equiatomic Ti_1.4_Nb_0.6_Ta_0.6_Zr_1.4_Mo_0.6_ bio-HEA [41]. An excellent degradation resistance in a simulated physiological environment of bio-HEA MoNbTaTiZr alloy compared to 304 stainless steel (304 SS) was also reported in the literature by Shittu et al. [42]. For equiatomic bio-HEA, the presence of dual-BCC phases was confirmed. Additionally, studied high entropy alloy exhibited higher hardness, reduced Young’s modulus, wear resistance and biocompatibility in comparison to 304 SS. Taking the above into account, equiatomic MoNbTaTiZr high entropy composition exhibited promising applicability due to improved service life and low toxicity for the human body [42].

The literature-reported data also revealed Hf-containing high entropy alloys for biomedical applications. Bio-corrosion and biocompatibility measurements of TiZrHfNbTa high entropy alloy compared to Ti6Al4V alloy were reported by Yang et al. [43]. For studied HEA composition, spontaneously passivating behavior with a low passive current density, low corrosion rate and high electrochemical impedance has been revealed. In vitro studies of TiZrHfNbTa showed good adhesion, viability and proliferation of pre-osteoblasts, which indicated good biocompatibility properties of the studied high entropy material [43]. Independent studies performed and reported by Yuan et al. also revealed low magnetic susceptibility of TiZrHfNbTa high entropy alloy [44]. Motallebzadeh et al. in 2019 also proposed two high entropy compositions containing Hf for biomedical applications (TiZrTaHfNb and Ti_1.5_ZrTa_0.5_Hf_0.5_Nb_0.5_) [45]. A single-phase BCC solid solution with small differences in lattice parameters was confirmed for both alloys. Mechanical properties revealed high yield strength of Ti-enriched composition compared to equiatomic composition. However, differences in hardness and reduced Young’s modulus were observed for both alloys. Moreover, studied alloys exhibited good corrosion resistance but smaller wettability in comparison to Ti6Al4V alloys [45]. Corrosion resistance measurements of three high entropy compositions TiTaHfNb, TiTaHfNbZr and TiTaHfMoZr, designed as medical implants, were described by Gurel et al. [46]. Corrosion resistance measurements were performed for long-term studies by immersion in simulated body fluid (SBF) and artificial saliva (AS) mixtures. Improvement of corrosion resistance reduced ion release, and better surface properties were obtained due to Zr and Nb alloying elements. For the TiTaHfMoZr sample, a significant amount of ion release upon immersion in both media was observed due to an inhomogeneous microstructure leading to the formation of dendritic structures. However, passive layers on all sample surfaces ensure corrosion resistance in long-term applications [46].

It is worth emphasizing that, up to now, the six-elemental high entropy alloys containing all of the six proposed chemical elements (TiTaNbZrHfMo) described in the current manuscript were studied mainly for refractory and turbine engine applications. For turbine blade applications, HEAs were obtained via vacuum arc melting, and the presence of BCC disordered solid solution was revealed [47]. For studied alloys, microstructural segregation to dendritic and interdendritic regions was observed. Mechanical property measurements confirmed high yield and ultimate strength values. Additionally, the strong solution strengthening effect was observed, which could be an advantage for high-temperature applications [47]. Independent studies reported by Bhandari et al. used thermodynamic calculations to investigate the structural, mechanical and thermodynamic properties of HfMoTaTiZr high entropy alloy [48]. Based on the calculated phase diagram, it can be revealed that the stable BCC phase was formed in the temperature range 1000–2100 K. On the other hand, it was also shown that hexagonal closed packed (HCP) and Laves phases (C15) could be formed below 800 K. Chen et al. for TaNbHfZrTi HEAs in an as-homogenized state also revealed the possibility of the presence of HCP and BCC solid solutions. Phase formation possibility is closely correlated with temperatures and times of the homogenization process [49]. The influence of Mo single element on the microstructure and mechanical properties of HfMo_x_NbTaTiZr (where x = 0, 0.25, 0.5, 0.75, 1) high entropy alloys was investigated and reported by Juan et al. [50]. For samples obtained via vacuum arc melting, single-phase BCC solid solutions have been revealed. Additionally, it was also confirmed that increasing Mo addition decreases the lattice parameters. Increased Mo content also improved mechanical properties such as yield strength and Young’s modulus. A strong solid solution strengthening effect increasing with the increase of Mo concentration was observed. It is also worth mentioning that the solid solution strengthening effect of Mo addition was also confirmed and reported by Tseng et al. [47].

Based on the above-described literature data, the presented work aims to design and to obtain novel high entropy Ti_20_Ta_20_Nb_20_(ZrHf)_20−x_Mo_x_ (where: x = 0, 5, 10, 15, 20 at.%) alloys composed of biocompatible elements: Ti, Nb, Ta, Zr, Hf and Mo. Additionally, the presented work’s second aim is to determine the influence of the ZrHf/Mo ratio on the phase formation, microstructure, mechanical properties and corrosion resistance in a simulated body fluid environment. The studies were undertaken in order to fill the knowledge gap regarding biocompatible HEA in the vicinity of the atomic equilibrium composition mainly studied by other researchers. We decided to keep the TiTaNb atomic ratio unchanged due to the previously reported promising literature data on the ternary Ti alloys. As was proved, the literature-reported HEA compositions containing all six elements ensured high potential and biomedical application ability. Moreover, the influence of a single element—Mo addition—on the microstructure and mechanical properties was studied only for high-temperature applications by Tseng and Juan et al. [47,50]. The additional novelty of the current work is that the HEAs were produced from the powders not like previously from bulk elements. The results in the presented work could develop the current state of knowledge about biomedical high entropy alloys containing the above refractory, biocompatible elements.

## 2. Materials and Methods

High entropy Ti_20_Ta_20_Nb_20_(ZrHf)_20−x_Mo_x_ (where: x = 0, 5, 10, 15, 20 at.%) alloys were designed and produced. The calculations of literature-reported thermodynamic parameters were carried out for phase formation predictions after solidification and to prove the classification of the designed alloy to the HEA group. As is well known for solid solution (SS) formation in binary alloys, Hume–Rothery (H-R) rules are fundamental. However, for HEAs, some H-R rules were also adopted in phase formation predictions. Due to multi-component chemical compositions of high entropy alloys, these rules have been developed by other important factors such as the temperature factor. In thermodynamic calculations, atomic size mismatch—δ and mixing enthalpy—ΔHmix of all alloying elements was taken into account, respectively [51]. In phase prediction, alloying elements’ electronegativity—Δχ—also significantly influences SS formation after solidification [52]. It was also reported that the valence electron concentration (VEC) should also be considered in phase formation [53]. Temperature factors such as melting points of alloying elements—T(m)i and an alloy’s empirical melting point—T(m) strongly influence solid solution formation [54]. A detailed description of the above-mentioned thermodynamic parameters was presented in [55]. The physical properties of all alloying elements and calculated thermodynamic parameters for studied alloys are presented in Table 1 and Table 2, respectively.

Samples were prepared from air plasma sprayed (APS) elemental powders of Ti (purity 99.9%, particle size < 90 μm), Ta (purity 99.9%, particle size < 100 μm), Nb (purity 99.9%, particle size 70–180 μm), Mo (purity 99.5%, particle size < 90 μm) provided by Kamb Import-Export, Warsaw, Poland and Zr (Atlantic Equipment Engineers, Upper Saddle River, NJ, USA—purity 99.5%, particle size < 250 μm). Hf powder was obtained by mechanical grinding from the bulk rod of purity < 99%. Elemental powders were blended for 72 h to achieve homogenous particle distribution. Green compacts from as-blended powders of 10 mm diameter were compacted under the pressure of ~3330 MPa. As-pressed green compacts were produced via a vacuum arc melting (VAM) technique in an Ar atmosphere (chamber pressure of 1.2 bar). High-purity Ti-getter was used for residual gas capturing in a vacuum chamber. Alloys were preliminarily melted and mixed up in a liquid state for 120 s. Furthermore, obtained VAM buttons were flipped over and re-melted four times concerning homogeneity of chemical compositions. For all studied alloys, no further thermo-mechanical treatment was performed. Subsequently, the samples were embedded into resin and ground using SiC grinding papers (grit from 320 to 2400). The samples were further polished using diamond suspensions with particle sizes from 6 μm to 1 μm. Final polishing was performed using colloidal silica oxide (SiO_2_) suspension with 0.04-μm particle size.

The X-ray powder diffraction (XRD) measurements were carried out using a Panalytical Empyrean diffractometer (Malvern Instruments, Malvern, UK) with Cu anode (with a wavelength of 1.54056 Å) working at an electric current of 30 mA, voltage of 40 kV, and equipped with a PIXcell^3D^ ultra-fast solid-state hybrid detector (Malvern Instruments, Malvern, UK). The X-ray diffraction measurements were performed in an angular range of 2θ = 20–110° with 0.02° step in Bragg–Brentano geometry (θ–θ scan technique), and the time count was 1200 s for each point at room temperature T ≈ 300 K. Determination of unit cell parameters was carried out by Powley refinement in FullProf open access computer software [59].

The scanning electron microscope (SEM) microstructure analysis was performed using the JEOL JSM-6480 (JEOL Ltd., Tokyo, Japan), working with the accelerating voltage of 20 kV and equipped with the energy-dispersive X-ray spectroscopy (EDS) IXRF detector (IXRF, Austin, TX, USA). The microstructure of studied high entropy alloys was also studied using Thermo Fischer G2 200 Themis (Thermo Fisher Scientific, Waltham, MA, USA) scanning–transmission electron microscope (STEM) working at 200 keV acceleration voltage, equipped with a spherical aberration image corrector. The TEM images were recorded using Thermo Fisher 4 k × 4 k CETA 16 CMOS camera (Thermo Fisher Scientific, Waltham, MA, USA). Z-contrast images in STEM mode were carried out using a high-angle annular dark-field (HAADF) detector from the Fischione (E.A. Fischione Instruments, Inc., Pennsylvania, PA, USA). Composition maps were measured in the STEM with a 4-segmented Super-X EDX System (FEI, Eindhoven, The Netherlands). EDS spectra were collected and evaluated by the Velox software (version 3.3, FEI, Eindhoven, The Netherlands). Thin lamellas for TEM observations were cut out using the focused ion beam (FIB) technique from the central part of the VAM buttons. FIB lamellas were cut using ThermoFisher Scios 2 Dual Beam microscope (Eindhoven, The Netherlands) equipped with an EasyLiftTM nanomanipulator. During gallium (Ga) ion thinning, the current of the ion gun was changed to reduce the amorphization possibility of studied alloys. Additionally, platinum (Pt) deposition was used to protect the surface of TEM thin lamellas. TEM Selected Area Electron Diffraction (SAED) pattern was indexed using the ElDyf computer software (version 2.1, Institute of Materials Engineering, Chorzów, Poland).

Microhardness measurements were carried out using MicroVickers tester 401MVD (Wilson Instruments, Massachusetts, MA, USA) equipped in ~136° pyramidal Vickers tip, under the load of 1 kN (HV 1) and dwell time of 10 s. Nanohardness and reduced Young’s modulus measurements were performed using Hysitron TI 950 Triboindenter (Bruker, Billerica, MA, USA) equipped in Berkovich tip with a total included angle ~142°. Nanoindentation measurements were carried out using a maximum load of 1000 μN with the load function composed of 5 s loading and unloading segments, separated by a dwell time of 2 s.

For corrosion resistance measurements, disc-shaped samples of the studied high entropy alloys were grounded with 80 to 2500# grit SiC paper and finally polished using colloidal silica oxide (SiO_2_) suspension. The in vitro corrosion resistance of the studied materials was investigated in Ringer’s solution (8.6 g/L NaCl, 0.3 g/L KCl, 0.48 g/L CaCl × 6H_2_O) deaerated with argon (Ar, 99.999%) at 37(1) °C. A three-electrode electrochemical cell was used where the studied materials were the working electrode (WE). The Pt mesh was used as a counter electrode (CE), and the saturated calomel electrode (SCE) with a Luggin capillary was the reference electrode (RE). The electrochemical measurements were carried out using a Metrohm/Eco Chemie Autloab PGSTAT30 Potentiostat/Galvanostat Electrochemical System (Herisau, Switzerland). Before electrochemical measurements, the WE electrodes were depassivated at −1.2 V vs. SCE for 10 min. Open circuit potential (E_OC_), potentiodynamic polarization and electrochemical impedance spectroscopy (EIS) methods were applied. The E_OC_ was registered for 2 h. The EIS measurements were performed at E_OC_ in the frequency range of f = 50 kHz–1 mHz. Ten frequencies per decade were scanned using a sine-wave amplitude of 10 mV. Anodic polarization curves were registered potentiodynamically at a sweep rate of v = mV s^−1^ in the potential range from E_OC_ minus 150 mV till break down potential occurred.

## 3. Results and Discussion

### 3.1. Microstructure Analysis of Initial Powders Elements

**Figure 1:**
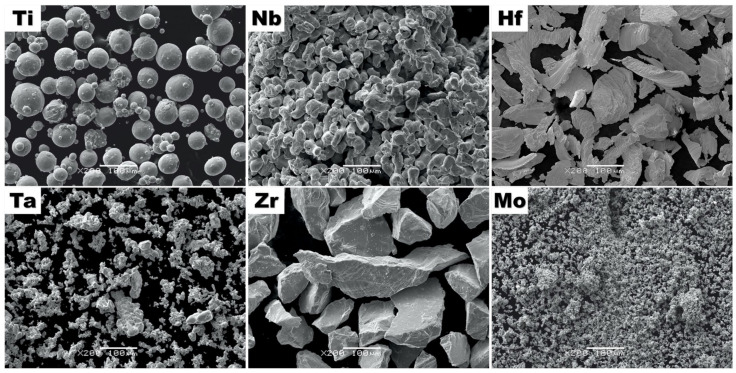
Secondary electron contrast (SEI) microstructure images of Ti, Ta, Nb, Zr, Hf and Mo initial powders.

Microstructure analysis of initial powders was performed using the scanning electron microscopy (SEM) technique. Secondary electron imagining mode images (SEI) of elemental powders are presented in Figure 1.

Microstructure analysis revealed a closely spherical shape of Ti powder with different particle sizes. Additionally, for Nb, Ta and Mo powders, the presence of agglomeration was also observed. On the other hand, coarse particle sizes for Zr and Hf were obtained.

### 3.2. XRD Phase Analysis of Studied High Entropy Alloys

Phase analysis was performed based on the recorded X-ray diffraction patterns. For the Mo_0 sample single BBC structure was observed, whereas, for all Mo containing high entropy compositions, X-ray diffraction revealed the presence of two-phase–dual-BCC structures. No additional diffraction peaks were observed. For all recorded diffraction patterns, Powley refinement was performed in order to determine the lattice parameters of the studied alloys (see Table 3). The slight differences between lattice parameters of BCC1 and BCC2 solid solutions were revealed. It is worth emphasizing that similar phenomena were observed for other high-entropy alloys described by the literature-reported data [38,39,45].

**Figure 2:**
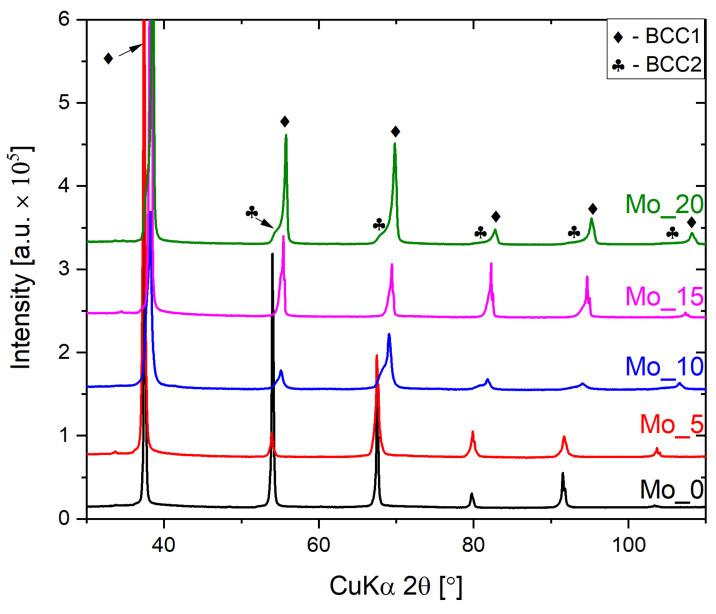
Measured X-ray diffraction patterns for all studied high entropy alloys.

A comparison of X-ray diffraction patterns recorded for all studied high entropy alloys is presented in Figure 2. As can be observed, the diffraction peaks corresponding to the BCC2 phase arise with increasing Mo contents and are present in the vicinity of the intense diffraction peaks corresponding to the BCC1 phase.

**Figure 3:**
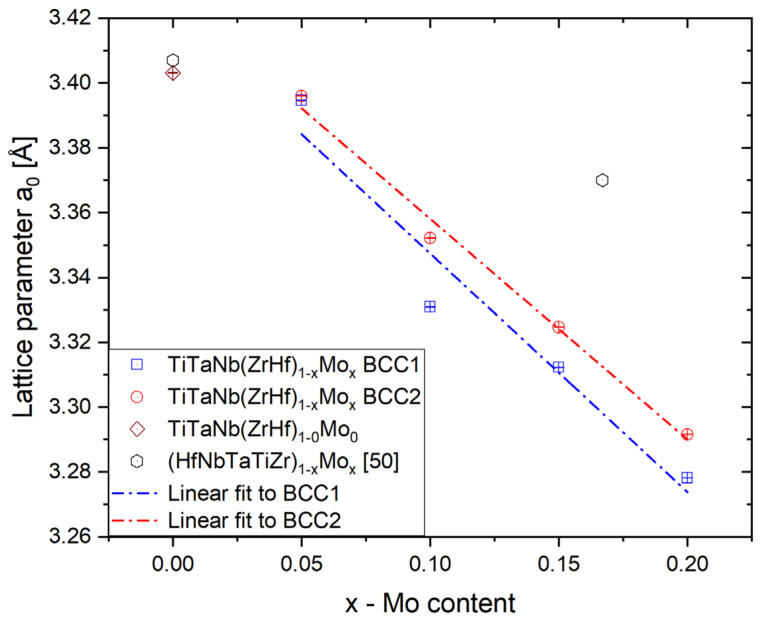
The changes of lattice parameters of dendritic (BCC1) and interdendritic (BCC2) phases with different Mo content for all studied high entropy alloys.

The performed Powley refinement showed that the lattice parameters for both BCC phases decreased with increased Mo addition. As shown in Figure 3, the determined lattice parameters for both observed phases follow a linear decline with increasing Mo content. The determined lattice parameters linear coefficient for both phases are as follows: −0.68(5) Å for BCC1 and −0.74(12) Å for BCC2, respectively.

For the alloy without Mo addition, the obtained lattice parameter stays in good agreement with the literature reported HfMo_x_NbTaTiZr (x = 0.0) high entropy alloy presented in reference [50] (marked as a circle in Figure 3). The lattice parameters of the HfMo_x_NbTaTiZr single-phase BCC solid solution also decreased with increasing Mo content. The lattice parameter of the HEA presented in this work also decreases but with significantly stronger dependence due to variation in the Mo content and change in the ZrHf/Mo ratio. The variation of the lattice parameters of both BCC1 and BCC2 phases could be correlated with enrichment in prominent atomic radii elements (see Table 1 and Table 4).

### 3.3. SEM Microstructure Analysis of Studied High Entropy Alloys

**Figure 4:**
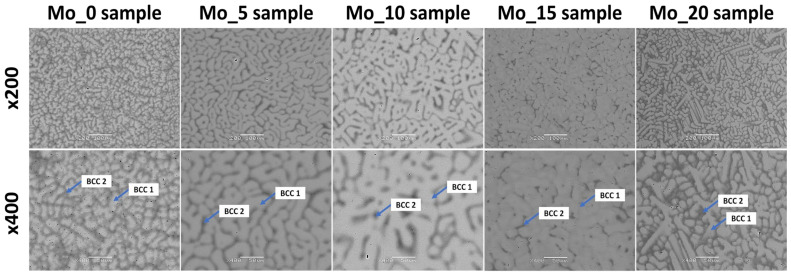
Backscattered electron contrast imaging mode (BSE) microstructure images of studied high entropy alloy compositions with assigned BCC solid solutions corresponding to dendritic and interdendritic regions.

The microstructure analysis using the SEM technique confirmed the presence of two phases (dendritic and interdendritic structures) with different chemical compositions. These regions were formed after solidification based on melting point differences due to elemental segregation [38,39,47,50]. For all studied alloys, based on the measured chemical composition, the calculated melting temperature of the BCC1 phase is higher than BCC2 (see Table 4). Such observation stays in good agreement with the observed microstructure. The crystallization process of the higher melting phase starts first and promotes the creation of a dendritic structure. SEM observations are in good agreement with the XRD phase analysis that revealed dual-BCC solid solutions, which can be attributed to dendrites and interdendritic regions. References [38,39] also indicate the possibility of the presence of dual-BCC structures based on the microstructure analysis for TiTaNbZrMo biomedical high entropy alloys with similar chemical composition. The observed microstructure presented in the current work stays in high agreement with the previous literature-reported results. Recorded SEM images in backscattered electron contrast imaging mode (BSE) are presented in Figure 4. According to the performed phase analysis and literature data from references [38,39], dendritic and interdendritic regions were denoted as BCC1 and BCC2, respectively. As can be seen in Figure 4, the interdimeric BCC2 phase separates from each other dendritic grains of the BCC1 phase. Based on the grain boundary wetting theory described by J. W. Cahn [60] and further studied by Straumal et al. [61,62,63] such observation indicates that during the solidification process the molten metal completely separates the majority of BCC1 primary grains from each other. Grain boundaries of BCC1 phase were completely wetted by the mold.

For the chemical composition measurements, the EDS technique was used. EDS chemical composition analysis confirmed the presence of all principal elements with various elemental segregations for BCC1 and BCC2 phases, respectively (see Table 4).

For all studied alloys, the chemical analysis revealed the presence of all alloying elements; nevertheless, the chemical composition is slightly different from the nominal one and varies from alloy to alloy. EDS chemical composition analysis revealed that the dendritic structure (BCC1 phase) was depleted in Hf and Ta and enriched in Ti and Nb alloying elements for studied high entropy alloys. On the other hand, for the BCC2 phase corresponding to interdendritic regions, enrichment of Ti, Nb and Zr chemical elements and depletion in Hf and Ta was observed. It is worth emphasizing that elemental segregation for all studied high entropy alloys was closely correlated with the calculated melting points of observed phases (see Table 1) and stayed in good agreement with the data reported in references [38,39,47].

### 3.4. TEM and STEM Microstructure Analysis of Studied High Entropy Alloys

**Figure 5:**
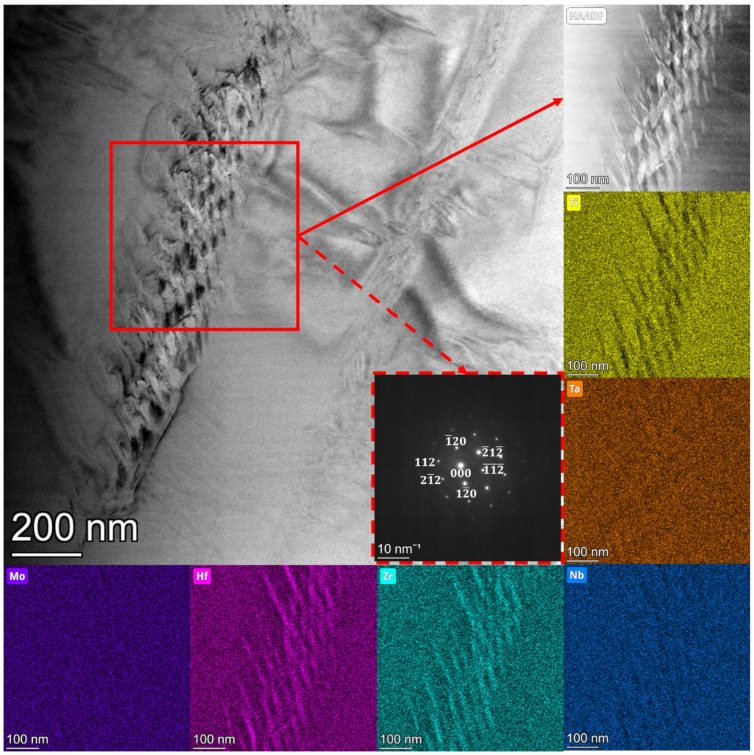
Bright-field and indexed SAED image of Hf-Zr recorded in TEM mode with the corresponding STEM high-angle annular dark-field image (HAADF), and elemental distribution maps for Mo_15 sample.

Microstructure analysis was also performed using scanning–transmission electron microscopy (STEM) techniques. For all studied samples, the microstructure was quite similar. The detailed microstructure analysis showed the presence of a small amount of lamellar-shaped precipitates. The precipitates were mostly visible in the sample Mo_15; due to that, in the current manuscript, we limit ourselves to presenting the results recorded for the Mo 15 sample. The bright-field (BF), selected area electron diffraction (SAED), HAADF and elemental distribution map images are presented (Figure 5). STEM elemental distribution maps for Ti, Nb, Ta and Mo revealed the homogeneity distribution of all these elements in the studied area. The observed lamellar-shaped precipitates are enriched with Hf and Zr, as can be seen on Zr and Hf elemental distribution maps (see Figure 5). The SAED indexed pattern is in good agreement with the Hf-Zr hexagonal (HCP) phase. The amount and size of the precipitates were not enough to be seen on the recorded XRD diffraction patterns. Nevertheless, similar precipitates were observed for the HfNbTaTiZr high entropy alloy studied by Chen et al. [49]. The precipitates were dissolved in the homogenization process, up to 700 °C, and were not observed in XRD patterns. It is highly probable that Hf-Zr precipitates observed in the studied alloys can be dissolved during homogenization. Further studies of the thermo-mechanical process of the studied alloys will be undertaken in forthcoming work.

### 3.5. Corrosion Resistance Measurements of Studied High Entropy Alloys

In the in vitro studies, the E_OC_ measurements were carried out for 2 h until the ionic-electron equilibrium related to the formation of a double electrical layer at the electrolyte/oxide layer interface was stabilized. The registered values of the E_OC_ for tested alloys can be treated as the approximate value of corrosion potential (E_cor_), and they varied depending on the Mo content in the studied high entropy alloys. The E_OC_ value, registered for studied HEAs, shifted toward the positive values may indicate better corrosion resistance in comparison to alloy without molybdenum addition—Mo_0 sample (see Table 5). Differences in the corrosion resistance of HEA electrodes may result mainly from the structure and chemical composition of self-passive oxide films present on the surface of the studied electrodes. The impedance measurements were performed under potentiostatic control at the determined E_OC_ values (Table 5).

**Figure 6:**
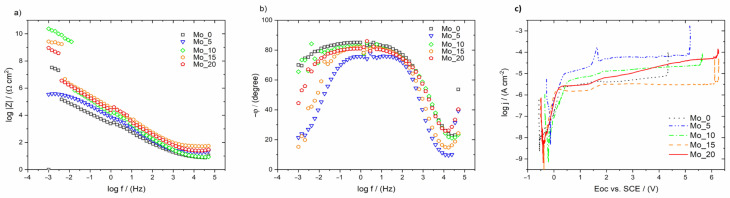
Bode diagram registered at E_OC_ (**a**) log |Z| = f(log f) and (**b**) φ = f(log f), and (**c**) anodic polarization curves for HEA electrodes exposed in Ringer’s solution at 37 °C.

The experimental EIS results in the form of Bode diagrams are presented in Figure 6a. The slope of log |Z| = f (log f) plots in the medium frequency range are close to −1 (Figure 6a). The increase in the value of the log |Z|_f→0.01Hz_ (Table 5) with higher Mo content in the alloys can be observed. Increasing the value of log |Z|_f→0.01Hz_ may indicate better resistance for pitting corrosion. The Bode diagrams displaying the φ in a function of the logarithm of the measuring frequency revealed a plateau in the range of medium frequencies, which indicates the passive protection of the studied electrodes (Figure 6b). Moreover, one time constant, visible on Bode diagrams, is characteristic of titanium and its alloys with a thin layer of self-passive oxide [64]. In any case, one can observe the deviation of the maximum values of φ from the ideal value of −90°. The high impedance values of log |Z|_f→0.01Hz_ (Table 5) and the phase angle, φ, close to −80°, are typical of a capacitive behavior of high corrosion resistant materials [65].

The higher impedance values (Figure 6a) and the broad plateau (Figure 6b) correspond to the more effective corrosion resistance. Corrosion processes were observed at low frequencies. The high dispersion of measured values can be observed in the Bode plots obtained for the studied electrodes at the low-frequency range (Figure 6). This kind of dispersion occurs when surface reconstruction takes place. In this case, it might indicate the “good healing” properties of studied HEAs and high resistance for pitting corrosion in Ringer’s solution [65]. This effect can be caused by the material’s characteristics and might indicate that the oxide present on their surfaces is a dielectric or semiconductor.

The potentiodynamic curves registered for the studied HEA electrodes in Ringer’s solution were typical for self-passivating materials (Figure 6c). The break-down potential of the oxide layers on the electrode’s surfaces was varied depending on the Mo content in the high entropy alloys. The highest break-down potential around 6.11 and 6.18 V vs. SCE was observed for Mo_15 and Mo_20 samples, respectively (Figure 6c). On the other hand, the lowest value (4.33 V vs. SCE) was registered for the Mo_0 sample. On the potentiodynamic curves, among the passive range could be distinguished a slight increase in current density in the potential range 1.5–2 V vs. SCE, which might be correlated with oxidation of the nonstoichiometric oxides during increasing potential values. It is well observed on the potentiodynamic curve registered for Mo_5 sample. The break-down potentials for studied high entropy alloys confirmed a high biomedical application ability in comparison to the break-down potential of the TiO_2_ oxide layer for pure Ti and Ti-based medical alloys (0.5–2.4 V) [66]. Moreover, for all studied Mo-containing HEAs, the break-down potential was higher compared to literature-reported data for widely used, commercial biomedical alloys such as cp-Ti Grade 2 alloy (1.48 V vs. SCE), Ti6Al4V alloy (1.53 V vs. SCE), ternary Ti6Al7Nb and Ti13Nb13Zr (1.38 V and 1.25 V vs. SCE, respectively) alloys and 316 L medical stainless steel (0.96 V vs. SCE) [67,68]. The studied HEAs with low Mo content exhibited lower break-down potential (Mo_0: 4.33 V and Mo_5: 5.15 vs. SCE) in comparison with binary Ti15Mo biomedical alloys (4.51 and 5.5 V vs. SCE) [69,70]. On the other hand, the obtained Mo-rich HEAs exhibited high break-down potential in comparison to various biomedical alloys such as binary Ti15Mo, Ti45Nb, Ti15Nb alloys (4.51, 0.28 and 0.45 V vs. SCE, respectively) and four-elemental, medium entropy TiNbZrTa biomedical alloy designed for orthopedic implant applications (>5.00 V vs. SCE) [71,72,73]. The break-down potential of NiTi SMAs (0.28 and 0.45 V vs. SCE), widely used in medicine, is also much smaller than determined for all obtained Ti_20_Ta_20_Nb_20_(ZrHf)_20−x_Mo_x_ HEAs [74,75].

### 3.6. Microhardness and Nanoindentation Measurements of Studied High Entropy Alloys

**Figure 7:**
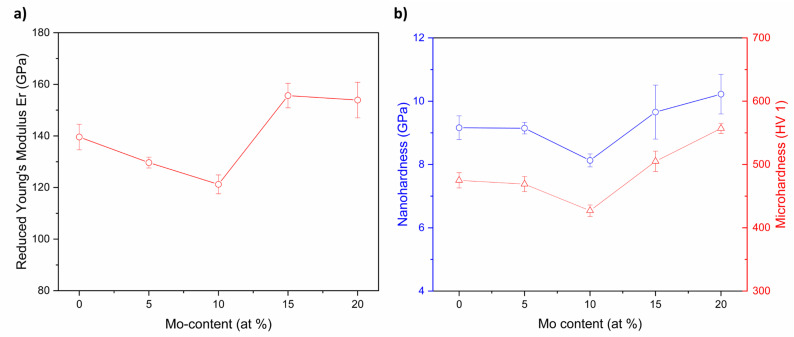
Reduced Young’s modulus (**a**), nanohardness and microhardness (**b**) values for different Mo content for studied high entropy alloys.

In order to determine the mechanical properties of the obtained alloys, microhardness measurements were carried out. Indentation with a micrometric tip was performed to obtain average microhardness values from both BCC1 and BCC2 phases (Table 6). Furthermore, nanoindentation measurements were performed to determine the differences in mechanical properties (nanohardness (H) and reduced Young’s modulus (Er)) with increasing Mo content in both phases of the studied high entropy alloys. After initial tests made for our two-phase high-entropy alloys, we concluded that their subtle dendritic structure prevents performing reliable measurements separately for both phases. Therefore, we decided to make a series of nanoindentations on grids composed of 10 × 20 points, with a 5 μm distance between them. The results collected for each alloy were then averaged to get representative mechanical characteristics. Our analysis utilized a standard procedure based on the Oliver–Pharr method [76]. Prior to the measurements, all samples were etched using 3% hydrofluoric acid (HF) + 3% nitric acid (HNO_3_) + distilled water (H_2_O) for 15 s solution to reveal their microstructure. The obtained results are presented in Table 6 and Figure 7.

Additionally, the dependence of the micro- and nanohardness and the reduced Young’s modulus of the Mo content is shown in Figure 7.

Obtained results confirmed the influence of the Mo content on micro- and nanohardness and on reduced Young’s for all studied high entropy alloys. Average H values decreased for alloys with Mo addition of 0 to 10%. It is highly probable that an increase in H and decrease in Er for samples containing more than 10% of Mo addition could be correlated with enrichment in large atomic radii elements in both high entropy alloys (see Table 1 and Table 4). Nanohardness measurements confirmed similar phenomena as for the microindentation one. A decrease of microhardness was observed for alloys with Mo addition of 0 to 10%. Moreover, the lowest microhardness was observed for the Mo_10 sample (HV 1 = 427(9)). On the other hand, further increase of microhardness was observed with the increase of Mo up to 20%. Additionally, obtained nanohardness and reduced Young’s modulus for all studied high entropy alloy compositions were compared with literature-reported conventional biomedical materials and biomedical HEAs (Table 7).

For all studied HEAs, reduced Young’s modulus was higher compared to humans’ tibial cortical (21.90 GPa and 25.20 GPa) and trabecular bones (15.90 GPa), which limits the application of studied alloys as metallic bone implants [77,78]. Nanohardness and reduced Young’s modulus for all studied materials were higher in comparison to literature-reported conventional biomedical alloy such as cp Titanium Grade 2 [79]. However, nanoindentation measurements exhibited the lower values of reduced Young’s modulus in comparison to 316 L medical stainless steel (196.77 GPa and 215.71 GPa) [45,82]. Mechanical property measurements of studied high entropy alloys exhibited higher nanohardness and Young’s modulus in comparison to literature-reported binary TiMo, TiNb and TiFe biomedical alloys [83,84,85]. Additionally, in comparison to ternary Ti-based TiMoNb, Sn-containing TiNbSn and Fe-containing TiFeTa biomedical alloys, nanoindentation measurements also confirmed higher values of nanohardness and reduced Young’s modulus for all studied Mo-containing HEAs [84,85,86]. Studied high entropy materials exhibited higher mechanical properties in comparison to literature-reported equiatomic and near equiatomic TiZrTaHfNb high entropy alloys [45]. On the other hand, for studied Mo-containing HEAs, the lower mechanical property values were confirmed compared to literature-reported ternary Ti-based TiNbZr, medium entropy TiZrNbTa and equiatomic, five-elemental TiZrNbTaMo biomedical high entropy alloys [38,81,87,88]. However, for all studied Mo-containing HEAs, nanohardness and reduced Young’s modulus were comparable to widely applied, literature-reported Ti6Al4V biomedical alloys [45,87].

It is highly probable that the improvement of mechanical properties of all studied high entropy alloys corresponds to the variety of chemical compositions. The changes in chemical composition could probably decrease nanohardness and reduce Young’s modulus closer to human bones. An increase of nanohardness and reduced Young’s modulus could also be correlated with the hafnium (Hf) chemical element in all studied high entropy alloys. The presence of Hf in microstructure contributes to the lattice distortion effect and improves the mechanical properties, as was reported in the literature for HfNbTaTiZr high entropy alloy by Zyka et al. [89]. However, if one would compare the present results to TiZrNbTaMo [38], it can be concluded that the presence of Hf could also decrease nanohardness and reduce Young’s modulus, respectively. Further studies of the influence of Hf addition on mechanical properties for similar chemical compositions will be undertaken.

## 4. Conclusions

Six-elemental high entropy Ti_20_Ta_20_Nb_20_(ZrHf)_20−x_Mo_x_ (where: x = 0, 5, 10, 15, 20 at.%) alloys were produced from elemental powders and by vacuum arc melting techniques. The influence of Mo/(ZrHf) ratio on phase and microstructure formation, mechanical properties and corrosion resistance in simulated body fluid environment in the as-cast state was analyzed. The chemical composition of all studied high entropy alloys contained all of the selected biocompatible elements.

For all studied Mo-containing HEAs, the presence of dual-BCC phases (dendritic and interdendritic) was observed by XRD and SEM. Small amounts of Hf-Zr hexagonal precipitates were revealed by STEM microstructure analysis and recorded electron diffraction patterns. The concentration of the precipitates was below the X-ray diffraction detection limit. The presence of similar precipitates was also reported in the literature for HfNbTaTiZr high entropy alloy in intermediate temperatures (up to 700 °C). XRD Powley refinement revealed slight differences of lattice parameters between dendritic and interdendritic phases. Analysis showed that the lattice parameters depend on the Mo/(ZrHf) ratio in a linear manner. Moreover, lattice parameters stayed in good agreement with literature-reported XRD phase analysis for high entropy alloys with similar chemical compositions.

Mechanical property measurements confirmed promising biomedical application ability for all studied high entropy alloys due to lower hardness and reduced Young’s modulus compared to 316 L SS, ternary Ti-based TiNbZr biomedical alloys, medium entropy (TiZrNbTa) and high entropy TiZrNbTaMo biomedical alloys. On the other hand, higher nanohardness and reduced Young’s modulus compared to human bones limits the application of studied alloys as metallic implants. Additionally, the determined nanohardness and reduced Young’s modulus of all studied alloys were higher in comparison to conventional biomedical alloys such as cp-Ti, binary and ternary Ti-based biomedical alloys. Further, a decrease of nanohardness and reduced Young’s modulus of all studied Mo-containing HEAs could give a new area of biomedical application.

The corrosion resistance measurements in Ringer’s solution-simulated body fluid environment confirmed high biomedical application ability due to the presence of stable oxide layers. The measurements showed that Ti_20_Ta_20_Nb_20_(ZrHf)_20−x_Mo_x_ produced alloys where x = 15 and 20 at.% exhibit the highest break-down potential, which indicates their higher performance than other obtained alloys. The corrosion resistance of all studied Mo-containing HEAs was higher compared to biomedical alloys such as cp Titanium Grade 2, Ti6Al4V and 316 L SS alloys. Obtained results could be a background for future corrosion resistance measurements in different simulated body fluid environment solutions such as phosphate buffer saline (PBS) or Hanks’s solutions which could give the new area of biomedical application.

## Figures and Tables

**Table 1:** Physical properties of pure alloying elements: Crystal structure, atomic radius—r_i_, valence electron concentration—(VEC)_i_, Pauling’s electronegativity—χ_i_, and melting point—T(m)_i_. BCC: Body-centered cubic: HCP: hexagonal closest packed [56,57,58].

Element	Crystal Structure	r_i_ (Å)	VEC	χ_i_ (Pauling Units)	(Tm)_i_ (°C)
Ti	HCP	1.462	4	1.54	1660
Ta	BCC	1.430	5	1.50	3017
Nb	BCC	1.429	5	1.60	2468
Zr	HCP	1.603	4	1.33	1952
Hf	HCP	1.578	4	1.30	2233
Mo	BCC	1.363	6	2.16	2123

**Table 2:** Thermodynamic parameters calculated for the studied high entropy alloys: Atomic size mismatch—δ, mixing enthalpy—ΔH_mix_, mixing entropy—ΔS_mix_, electronegativity differences—Δχ, valence electron concentration—VEC, and an empirical melting point of an alloy—T(m).

Chemical Composition	Abbreviation	δ (%)	ΔH_mix_ (kJ∙mol^−1^)	ΔS_mix_ (J·(mol·K)^−1^)	Δχ (eV)	VEC	T(m) (°C)
Ti_20_Ta_20_Nb_20_(ZrHf)_20_	Mo_0	4.99	2.72	13.38	0.118	4.40	2249
Ti_20_Ta_20_Nb_20_(ZrHf)_17,5_Mo_5_	Mo_5	5.21	1.49	14.35	0.190	4.50	2278
Ti_20_Ta_20_Nb_20_(ZrHf)_15_Mo_10_	Mo_10	5.31	0.36	14.68	0.234	4.60	2307
Ti_20_Ta_20_Nb_20_(ZrHf)_12,5_Mo_15_	Mo_15	5.30	−0.67	14.72	0.264	4.70	2336
Ti_20_Ta_20_Nb_20_(ZrHf)_10_Mo_20_	Mo_20	5.17	−1.60	14.53	0.285	4.80	2366

**Table 3:** Unit cell parameters calculated from X-ray diffraction patterns after Powley refinement.

Studied Alloy	Phase	Lattice Parameter a_0_ (Å)
Mo_0	BCC	3.4031 (1)
Mo_5	BCC1	3.3946 (1)
	BCC2	3.3960 (1)
Mo_10	BCC1	3.3309 (2)
	BCC2	3.3522 (2)
Mo_15	BCC1	3.3122 (1)
	BCC2	3.3246 (1)
Mo_20	BCC1	3.2782 (1)
	BCC2	3.2916 (1)

**Table 4:** SEM Energy-Dispersive X-ray Spectroscopy (EDS) chemical compositions (at.%) and calculated melting temperature (T(m)) for all studied high entropy alloys for dendritic (BCC1) and interdendritic (BCC2) regions.

Studied Alloy	Elements	Ti	Ta	Nb	Zr	Hf	Mo	T (m) (°C)
Mo_0	Nominal	20	20	20	20	20	―	―
	BCC1	34.4 (0)	12.4 (2)	24.9 (1)	17.9 (2)	10.3 (1)	―	2135
	BCC2	33.7 (1)	7.5 (2)	22.7 (1)	25.2 (2)	10.9 (1)	―	2067
Mo_5	Nominal	20	20	20	17.5	17.5	5	―
	BCC1	14.7 (1)	23.2 (5)	23.5 (1)	16.6 (5)	17.1 (1)	4.9 (1)	2361
	BCC2	16.4 (2)	17.4 (3)	21.9 (1)	21.1 (3)	19.4 (4)	3.8 (1)	2278
Mo_10	Nominal	20	20	20	15	15	10	―
	BCC1	28.2 (1)	14.5 (1)	25.3 (1)	9.3 (1)	6.9 (1)	15.8 (1)	2285
	BCC2	33.8 (2)	6.5 (4)	21.3 (1)	20.5 (4)	8.9 (2)	8.8 (2)	2104
Mo_15	Nominal	20	20	20	12.5	12.5	15	―
	BCC1	32.1 (12)	19.0 (12)	19.1 (1)	7.3 (3)	6.7 (4)	15.8 (1)	2294
	BCC2	33.2 (12)	12.0 (9)	17.5 (1)	14.6 (4)	10.2 (5)	12.6 (2)	2190
Mo_20	Nominal	20	20	20	10	10	20	―
	BCC1	13.5 (1)	24.2 (3)	23.4 (1)	5.6 (2)	7.1 (2)	26.3 (1)	2503
	BCC2	20.0 (1)	10.2 (4)	19.0 (1)	19.8 (6)	15.5 (6)	15.5 (3)	2242

**Table 5:** Open circuit potential and the log |Z|_f→0.01Hz_ value registered for studied alloys with different Mo content.

Studied Alloy	E_OC_ vs. SCE (V)	log |Z|_f→0.01Hz_ (Ω∙cm^2^)	E_BD_ vs. SCE (V)
Mo_0	−0.423	4.93	4.33
Mo_5	−0.142	5.40	5.15
Mo_10	−0.195	5.84	5.57
Mo_15	−0.365	6.39	~6.11
Mo_20	−0.333	6.26	~6.18

**Table 6:** Average microhardness (HV 1), nanohardness (H) and reduced Young’s modulus (Er) for different Mo content for all studied high entropy alloys.

Studied Alloy	Microhardness HV 1	Nanohardness (H) (GPa)	Reduced Young’s Modulus (Er) (GPa)
Mo_0	475 (12)	140 (5)	9 (1)
Mo_5	469 (12)	130 (2)	9 (1)
Mo_10	427 (9)	121 (4)	8 (1)
Mo_15	505 (16)	156 (5)	10 (1)
Mo_20	557 (18)	154 (7)	10 (1)

**Table 7:** Comparison of nanohardness and reduced Young’s modulus for all studied alloys and literature-reported conventional biomedical materials and biomedical high entropy alloys.

Chemical Composition	Nanohardness (H) (GPa)	Reduced Young’s Modulus (Er) (GPa)	References
Ti_20_Ta_20_Nb_20_(ZrHf)_20_ (Mo_0)	9.00	140.00	present work
Ti_20_Ta_20_Nb_20_(ZrHf)_17,5_Mo_5_ (Mo_5)	9.00	130.00	
Ti_20_Ta_20_Nb_20_(ZrHf)_15_Mo_10_ (Mo_10)	8.00	121.00	
Ti_20_Ta_20_Nb_20_(ZrHf)_12,5_Mo_15_ (Mo_15)	10.00	156.00	
Ti_20_Ta_20_Nb_20_(ZrHf)_10_Mo_20_ (Mo_20)	10.00	154.00	
Human’s tibial cortical bone Osteonic lamellae	-	21.90	[77]
Human’s tibial cortical bone Interstitial lamellae	-	25.20	
Human’s trabecular bone	-	15.90	[78]
cp Titanium Grade 2 mechanical/abrasive	3.88	127.96	[79]
cp Titanium Grade 2 electropolished	2.31	68.56	
cp Titanium Grade 2 after magnetoelectropolished	1.47	26.93	
cp Ti as cast	3.10	125.00	[80]
Ti64 gyroid	5.35	134.52	
cp Ti produced by selective laser melting	3.61	122.00	
cp Ti	2.90	107.00	[81]
316 L stainless steel	3.68	196.77	[82]
316 L stainless steel	3.54	215.71	[45]
TiMo (after cold rolling in rolling direction)	5.29	127.00	[83]
TiMo (after cold rolling in transverse direction)	5.27	126.00	
TiMo (without Mo segregation)	4.94	115.00	
Ti8Fe	5.60	128.00	[84]
Ti_74_Nb_26_	3.61	75.10	[85]
Ti_72_Mo_2_Nb_26_	3.04	67.00	
Ti_70_Mo_4_Nb_26_	2.84	63.60	
Ti_68_Mo_6_Nb_26_	2.82	55.70	
Ti_66_Mo_8_Nb_26_	3.22	54.50	
TiNbZr as-cast	3.80	72.00	[81]
TiNbZr agened for 15 min	4.40	69.00	
TiNbZr agened for 1.5 h	4.40	66.00	
TiNbZr agened for 3 h	4.40	74.00	
TiNbZr agened for 6 h	5.20	81.00	
TiNbZr agened for 12 h	5.70	96.00	
TiNbZr agened for 24 h	6.20	97.00	
Ti_85_Nb_10_Sn_5_	3.40	80.00	[86]
Ti_82_Nb_13_Sn_5_	2.90	75.00	
Ti_79_Nb_16_Sn_5_	2.70	62.00	
Ti_75_Nb_20_Sn_5_	2.60	61.00	
Ti8Fe5Ta	5.00	119.00	[84]
Ti8Fe8Ta	4.40	107.50	
Ti9Fe9Ta	3.69	99.00	
Ti10Fe10Ta	3.45	92.00	
Ti6Al4V	3.62	131.62	[45]
Ti6Al4V	6.50	127.90	[87]
TiZrNbTa	―	132.00	
TiZrNbTa	4.60	116.00	[88]
Ti_1.5_ZrTa_0.5_Hf_0.5_Nb_0.5_	3.33	98.68	[45]
TiZrTaHfNb	3.48	114.13	
TiZrNbTaMo	―	168.00	[87]
TiZrNbTaMo Dendritic	6.40	161.00	[38]
TiZrNbTaMo Interdendritic	5.70	133.00	

## Data Availability

Data sharing not applicable.
